# Right Ventricular Outflow Obstruction Due to Metastatic Neuroendocrine Tumor

**DOI:** 10.7759/cureus.3261

**Published:** 2018-09-06

**Authors:** Akhil Sood, Simbo M Chiadika, Jamie M Everett, Jason Au, Julie Rowe

**Affiliations:** 1 Internal Medicine, University of Texas Health Science Center at Houston-Mcgovern Medical School, Houston, USA; 2 Internal Medicine, Division of Cardiovascular Medicine, University of Texas Health Science Center at Houston-Mcgovern Medical School, Houston, USA; 3 Pathology and Laboratory Medicine, University of Texas Health Science Center at Houston-Mcgovern Medical School, Houston, USA; 4 Diagnostic & Interventional Imaging, University of Texas Health Science Center at Houston-Mcgovern Medical School, Houston, USA; 5 Internal Medicine, Division of Oncology, University of Texas Health Science Center at Houston-Mcgovern Medical School, Houston, USA

**Keywords:** neuroendocrine tumor, heart failure, cardiac metastasis

## Abstract

Neuroendocrine tumors (NETs) are rare malignant tumors that arise from neuroendocrine cells of the gastrointestinal tract and often metastasize to the liver, lung, and bone. Cardiac metastasis of NETs is uncommon. We report a patient with a past medical history of a neuroendocrine tumor of the left femur presenting with signs and symptoms of new onset heart failure. Transthoracic echocardiogram and cardiac magnetic resonance showed a large mass within the right ventricle causing right ventricular outflow obstruction. A positron emission tomographic/computed tomographic scan (PET-CT) revealed increased uptake of fluorodeoxyglucose (FDG) activity within the right ventricle consistent with metastasis. Cardiac biopsy of the right ventricular mass revealed metastatic nonfunctioning neuroendocrine tumor. In view of the fact that it was a tumor that caused the right ventricular obstruction, the patient was started on chemotherapy with improvement of symptoms. This case highlights that in patients with a history of neuroendocrine tumor presenting with heart failure, cardiac metastasis should be included in the differential.

## Introduction

Neuroendocrine tumors (NETs) are rare, representing less than 1% of all malignant tumors. The most common sites of origin are the pancreas, intestine, and lung. Approximately 10% of all NETs have an unknown site of origin. These NETs often metastasize to the liver, lung, and bone [1]. Cardiac metastasis of NETs is uncommon. This is a unique case of a patient with a history of neuroendocrine tumor of the left femur presenting with new onset heart failure.

## Case presentation

A 68-year-old male with a past medical history of a neuroendocrine tumor (NET) of the left femur presented with progressive dyspnea, orthopnea, and lower extremity edema. Three years ago, the patient was found to have a mass on the left femur. Biopsy revealed poorly differentiated neuroendocrine carcinoma of unknown primary. He had undergone surgical resection of the left femoral tumor and above-knee amputation with adjuvant chemotherapy (cisplatin and etoposide) and radiation therapy. Routine surveillance imaging showed no evidence of malignancy. Chest computed tomographic (CT) and magnetic resonance imaging of the abdomen/pelvis with contrast were performed at three-month intervals for the first year followed by six-month intervals. The patient was in clinical remission for the last two years.

**Figure 1:**
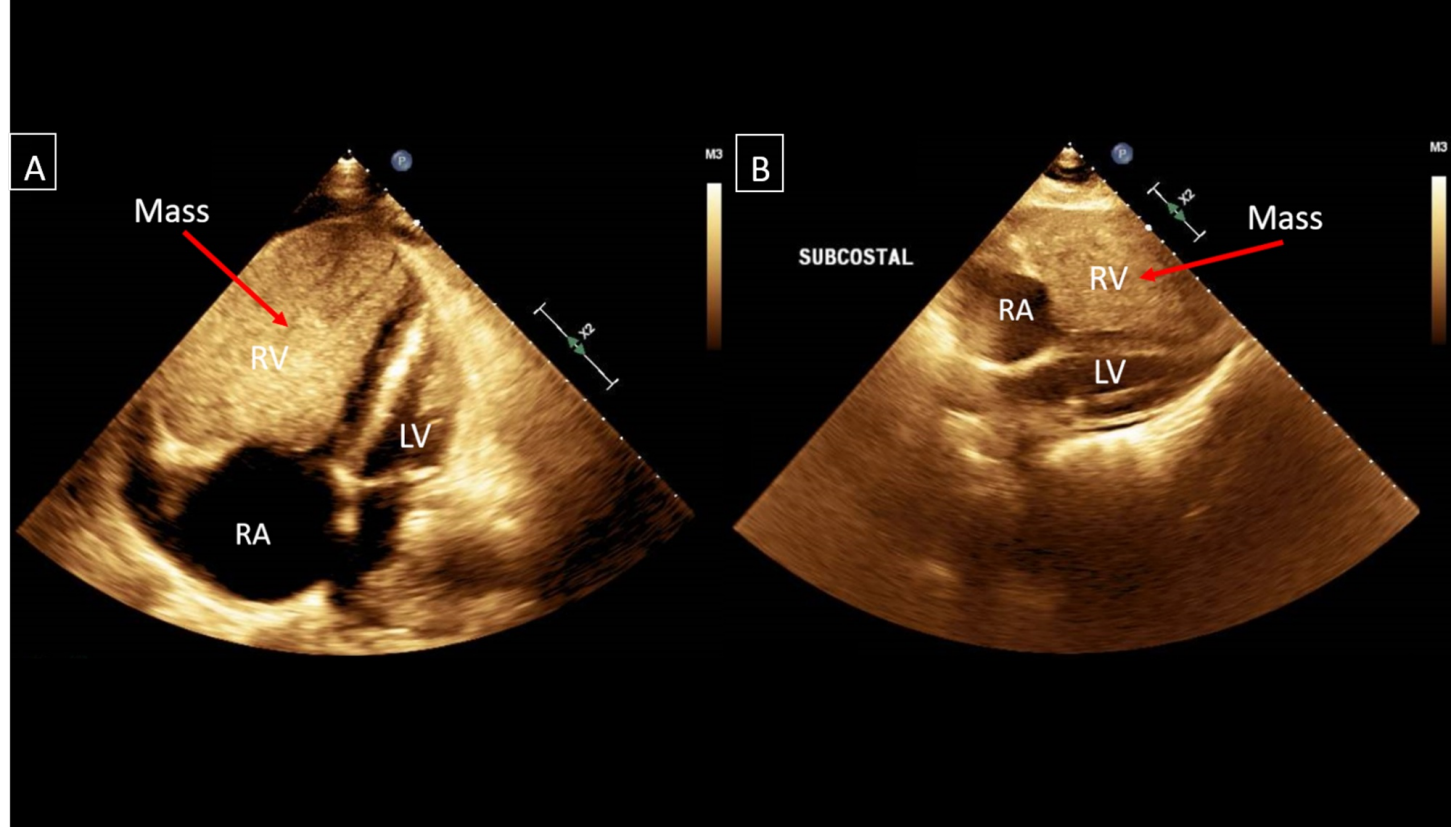
(A&B) Transthoracic echocardiogram in parasternal four-chamber view and subcostal view demonstrating a well circumscribed homogenous mass within the right ventricle measuring 8.10 X 6.54 cm and compressing the left ventricle. RA: Right Atrium; RV: Right Ventricle; LV: Left Ventricle

**Figure 2:**
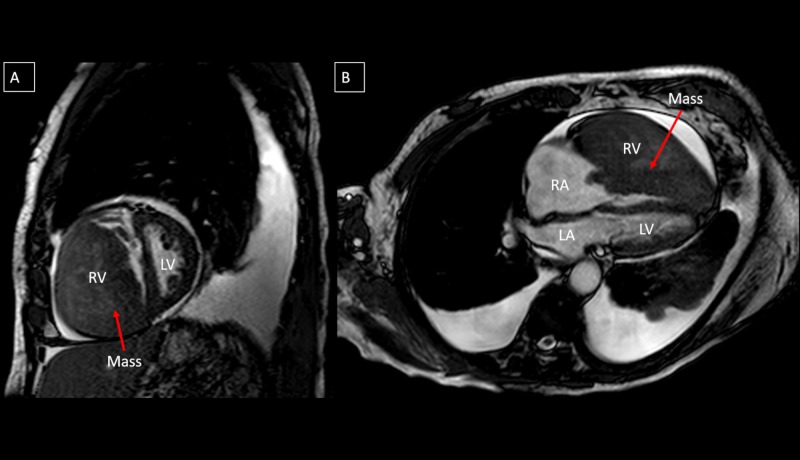
(A) Cardiac magnetic resonance in short axis view shows the mass extending from the right ventricular free wall both protruding through the tricuspid valve and obstructing outflow through the pulmonary outflow tract. (B) Four chamber view demonstrates the large mass obliterating the right ventricular cavity with extension into the right atrium. RA: Right Atrium; RV: Right Ventricle; LA: Left Atrium; LV: Left Ventricle

**Figure 3:**
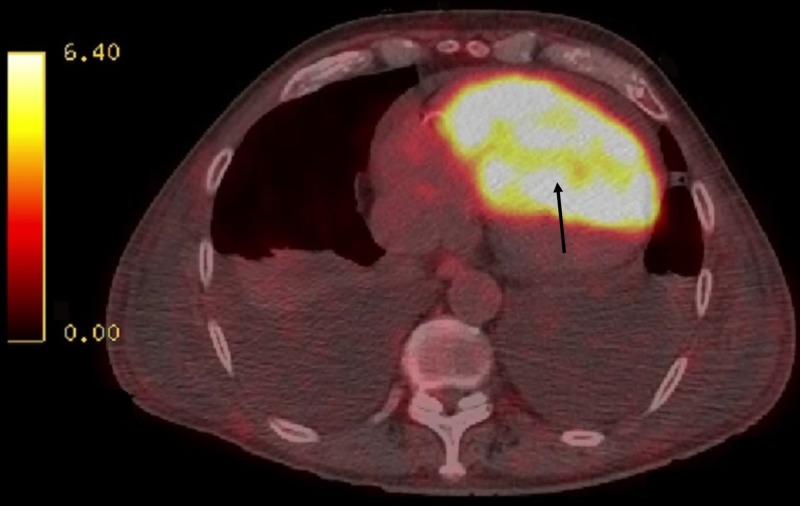
Positron emission tomographic/computed tomographic scan shows increased fluorodeoxyglucose activity within the right ventricular mass.

**Figure 4:**
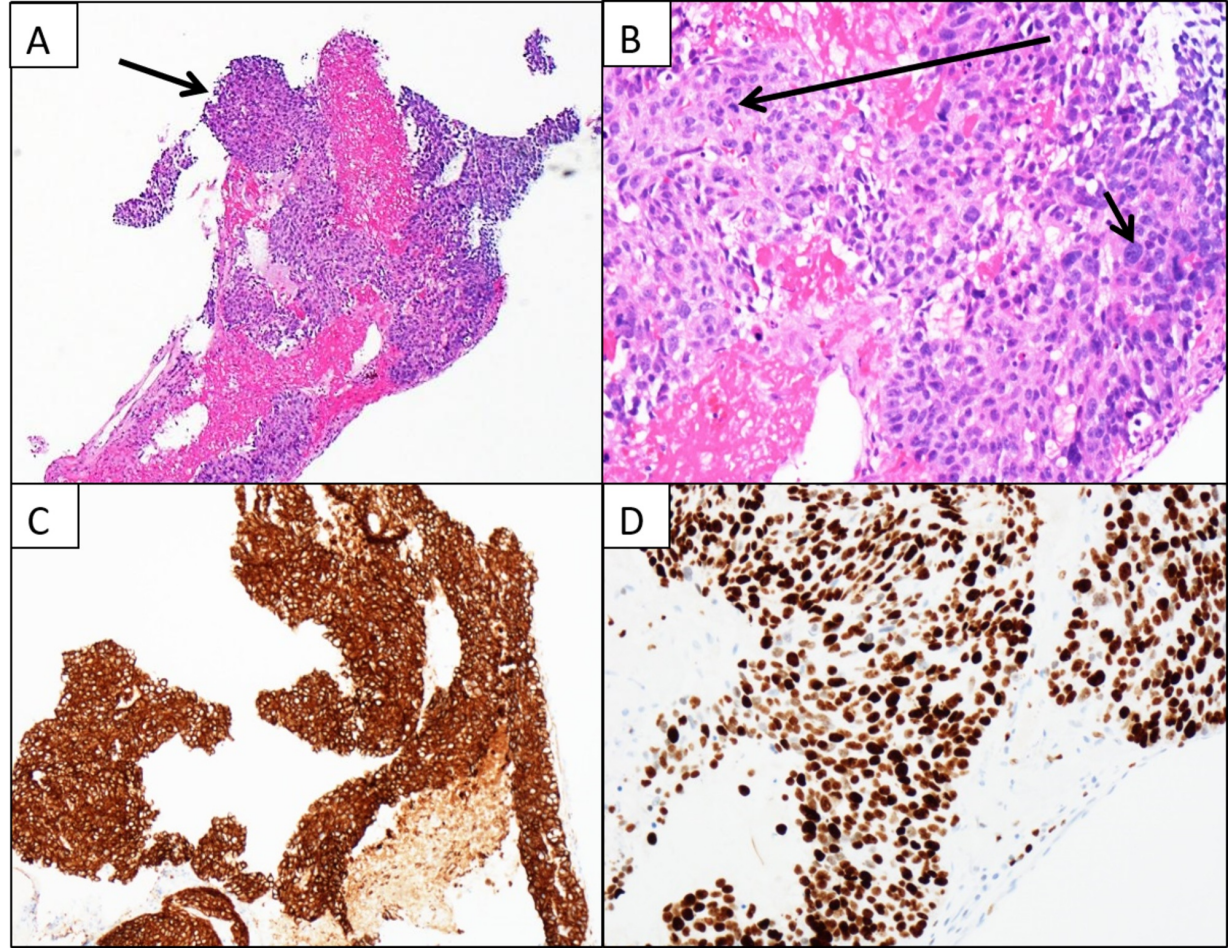
Histopathology and immunohistochemistry for metastatic tumor of the heart. (A) Low power field of a fragment of cellular tumor tissue admixed with blood. (B) High power field of a mitotic figure (long arrow) and a large tumor nucleus (short arrow). (C) Immunohistochemical stain for AE1/AE3 demonstrates cytoplasmic staining indicating epithelial origin of the carcinoma cells. (D) Immunohistochemical stain for KI-67 demonstrates nuclear staining in majority of the tumor cells consistent with a high proliferation rate.

On physical exam, his blood pressure was 119/76 mmHg, heart rate was 104 beats per minute, respiratory rate was 22 breaths per minute, and jugular venous pressure was elevated. Grade III/VI systolic ejection murmur was present at the left sternal border and rales at the lung bases. Chest X-ray revealed cardiomegaly and bilateral pleural effusions. A transthoracic echocardiogram revealed a large mass measuring 8.10 X 6.54 cm within the right ventricle causing right ventricular outflow obstruction, and the left ventricular ejection fraction was 60-65% (Figure 1). Cardiac magnetic resonance imaging confirmed the mass extending from the right ventricular free wall with compression of the left ventricle and dilated right atrium (Figure 2). A positron emission tomographic/computed tomographic scan showed increased standardized uptake value activity of 9.3 in the right ventricular mass (Figure 3). Cardiac biopsy of the right ventricular mass was consistent with metastatic neuroendocrine tumor (Figure 4). The tumor cells were negative for synaptophysin and chromogranin A but positive for CDX2, a marker for neuroendocrine tumor of unknown primary [2]. In view of the tumor that caused impairment in the right ventricular filling and congestive heart failure (CHF), the patient received chemotherapy (doxorubicin and cyclophosphamide) with improvement in the right ventricular failure.

## Discussion

Neuroendocrine tumors can be functioning or nonfunctioning depending on the presence or absence of hormonal hypersecretion. Functioning neuroendocrine tumors often release various vasoactive substances (serotonin, histamine, and bradykinin). Typical presentation includes flushing, wheezing, and diarrhea [3]. Heart failure in a patient with functioning NET is due to associated carcinoid heart disease. This involves secretion of vasoactive substances, leading to plaque-like deposits of fibrous tissue on cardiac valve cusps and leaflets. Our patient had a nonfunctioning NET, negative for all biomarkers including 5-hydroxyindoleacetic acid (5-HIAA) and chromogranin [4-5]. Congestive heart failure was due to intra-cardiac metastasis by the tumor.

Metastatic spread to the heart is more common than primary heart tumors. The most common tumors to metastasize to the heart include leukemia, lymphoma, and melanoma. Depending on the size and severity of cardiac metastases, symptoms can vary. This includes intracardiac tumors causing conduction deficits and cardiac arrhythmias. Pericardial involvement can lead to inflammation and effusion of the pericardium [3]. In our case, a large tumor within the right ventricle caused outflow obstruction, leading to congestive heart failure.

Generally, treatment for cardiac metastases of NETs is palliative. It is based on the primary tumor [3]. Our patient was initially treated with cisplatin and etoposide for NET of the left femur with unknown primary. Due to this exposure, the patient was switched to second line treatment cyclophosphamide and doxorubicin [6]. The patient tolerated chemotherapy well with improvement of symptoms. Radiation therapy was not indicated due to the risk of fibrosis of lungs and myocardium. Surgical resection was not performed given the high comorbid risk [3].

There have been only a few reports of neuroendocrine tumor metastasis to the heart. Cardiac metastasis is often asymptomatic and detected on autopsy [7]. Nonfunctioning neuroendocrine tumor presenting with congestive heart failure has not been previously reported. This is a unique case with radiologic and pathologic evidence suggestive of a nonfunctioning NET causing congestive heart failure.

## Conclusions

In a patient with a history of functioning neuroendocrine tumor, heart failure is typically due to carcinoid heart disease. Patients with nonfunctioning NET can present with congestive heart failure, mimicking carcinoid heart disease. In patients with a history of NET, presenting with heart failure, the clinician should also consider the possibility of cardiac metastasis.
